# Assessment of Quality of Life in Chronic Otitis Media Patients Undergoing Type I Tympanoplasty

**DOI:** 10.7759/cureus.83060

**Published:** 2025-04-27

**Authors:** Deepak K Gupta, Vishwadeep Singh

**Affiliations:** 1 Otolaryngology - Head and Neck Surgery, Vardhman Mahavir Medical College and Safdarjung Hospital, New Delhi, IND

**Keywords:** comot-15, improvement in hearing and mental health, mental health post tympanoplasty, quality of life post tympanoplasty, type 1 tympanoplasty

## Abstract

Introduction: Chronic otitis media (COM) contributes to considerable morbidity, with hearing loss and tympanic membrane perforations negatively affecting quality of life (QOL). This study aims to assess the outcomes following tympanoplasty in patients with unilateral mucosal COM, with a focus on health-related quality of life (HR-QOL) using the Chronic Otitis Media Outcome Test 15 (COMOT-15) questionnaire, in addition to evaluating audiological and surgical results.

Methodology: A prospective study of 70 patients undergoing Type I tympanoplasty was conducted from April 2023 to July 2024 in a tertiary care hospital in Delhi. Pre- and postoperative assessments included pure-tone audiometry (PTA) and COMOT-15 surveys. Graft uptake, hearing thresholds, and HR-QOL domains (symptoms, mental health, social function) were analyzed using paired t-tests and chi-square tests (IBM SPSS Statistics for Windows, Version 27 (Released 2020; IBM Corp., Armonk, New York)).

Results: Postoperatively, 85.7% achieved normal hearing (vs. 0% preoperatively; p = 0.001), with the mean air-bone gap improving from 28.85 ± 7.99 dB to 12.42 ± 7.26 dB (p = 0.001). Graft uptake was successful in 94.3%. COMOT-15 scores declined significantly (31.61 to 7.18; p = 0.001), with 85.7% reporting no QOL issues. Severe ear discharge reduced from 37.1% to 4.3% (p = 0.001), and psychological scores improved from 11.92 ± 1.67 to 0.77 ± 2.49 (p = 0.001). Hearing restoration was associated with mental health improvement (90.9% marked improvement; p < 0.001).

Conclusion: Type I tympanoplasty significantly restores hearing and HR-QOL in COM patients. The COMOT-15 tool effectively quantifies subjective benefits, highlighting the procedure’s dual clinical and psychosocial impact.

## Introduction

Chronic otitis media (COM) is a persistent middle ear inflammation characterized by ear discharge, hearing loss, and tympanic membrane perforation [1]. It is classified into mucosal and squamosal types, further divided into active and inactive states. COM is a significant public health concern, especially in developing countries, with an incidence of 3% to 57%, driven by factors like poor hygiene, overcrowding, and inadequate healthcare. Globally, it affects 65 million to 330 million people, causing severe hearing impairment in 60% and contributing to 2 million disability-adjusted life years (DALYs) and 28,000 deaths annually. Symptoms like foul-smelling discharge and hearing loss significantly impact quality of life, often leading to social withdrawal [2].

Management of COM (without ossicular erosion or fibrosis and without cholesteatoma) is tympanoplasty for repairing tympanic membrane perforations [3]. Graft materials like temporalis fascia and tragal or conchal cartilage are commonly used. Surgical outcomes depend on factors such as age, middle ear status, and eustachian tube function. While graft uptake and hearing improvement are traditional outcome measures, assessing health-related quality of life (HR-QOL) is equally important. The Chronic Otitis Media Outcome Test 15 (COMOT-15) is a validated tool that evaluates subjective improvements in symptoms, mental health, and social functioning [4].

This study was conducted to evaluate the impact of Type I tympanoplasty on the quality of life in patients with chronic otitis media. By comparing pre- and postoperative quality of life (QOL) using the COMOT-15 questionnaire, the study aims to capture the patient's satisfaction with recovery. Additionally, by exploring the association between hearing improvement and mental health, it seeks to highlight the broader benefits of tympanoplasty beyond surgical success.

## Materials and methods

Study design and setting

A prospective study was conducted over 15 months (April 2023 to July 2024) in the Department of Otorhinolaryngology at Vardhman Mahavir Medical College and Safdarjung Hospital, New Delhi, after Institutional Ethics Committee approval. Written informed consent was obtained from all participants.

Study population and sample size

Seventy patients diagnosed with unilateral mucosal COM requiring Type I tympanoplasty were included. The sample size was calculated based on a previous study of Shrestha et al. [5], with a 90% power and 5% significance level, accounting for a 20% loss to follow-up.

Inclusion and exclusion criteria 

Patients aged 18-50 years with unilateral mucosal COM were included. Exclusion criteria included sensorineural/mixed hearing loss, squamosal COM, ossicular discontinuity, comorbidities (e.g., diabetes, hypertension), complications of COM (such as cholesteatoma, ossicular erosion, mastoiditis, facial nerve palsy, meningitis, and abscess), and psychiatric illness.

Methodology

The patients were enrolled consecutively from the OPD of a tertiary hospital, employing purposive sampling. Patients underwent a detailed history, physical examination, and audiometric assessment using a MAICO MA51 audiometer (MAICO Diagnostics GmbH, Berlin, Germany). PTA for air and bone conduction at 500 Hz, 1000 Hz, 2000 Hz, and 3000 Hz was recorded under standardized conditions. The COMOT-15 questionnaire [6] was administered preoperatively to assess HR-QOL, which was validated and available in the local language. Permission to use the scale had been obtained.

Type I tympanoplasty was performed under local anesthesia using a postaural approach by the same surgical team. A temporalis fascia graft was placed medially to the malleus handle using the underlay technique. Postoperatively, patients received antibiotics, and those with ear discharge were treated based on culture sensitivity reports.

Follow-up assessments were conducted at three months to evaluate graft uptake and at four months to administer the COMOT-15 questionnaire and repeat PTA. Pre- and postoperative COMOT-15 scores and hearing outcomes were compared. The patients were contacted telephonically to ensure follow-up.

Statistical analysis 

Descriptive statistics (frequency, percentage, mean, standard deviation) and inferential statistics (Pearson chi-square test, paired t-test) were used. Psychological well-being scores (COMOT-15 questions 10-14) were similarly assessed. Data were analysed using IBM SPSS Statistics for Windows, Version 27 (Released 2020; IBM Corp., Armonk, New York). P< 0.05 was taken to be statistically significant.

Aim and objectives

The aim of the study was to assess the outcomes related to quality of life in patients with chronic otitis media who are undergoing Type I tympanoplasty. The primary objective is to compare the quality of life before and after the surgery using the COMOT-15 questionnaire. The secondary objective is to explore the association between improvement in hearing and mental health following the surgical intervention.

## Results

The current study was conducted among 70 patients undergoing Type I tympanoplasty. The mean age of the participants was 33.10 years, with a standard deviation of 8.93 years, ranging from 19 to 50 years. The majority (37, 52.9%) of the patients were male. Regarding perforation size, the majority of patients fell into the medium (38.6%, n = 27) and large (37.1%, n = 26) categories, while a smaller proportion had small perforations (21.4%, n = 15) or subtotal perforations (2.9%, n = 2). In terms of the affected side, the left side was more frequently involved (57.1%, n = 40) compared to the right side (42.9%, n = 30) (Table 1).

**Table 1:** Sociodemographic and clinical characteristics of patients (N = 70) Quantitative data are expressed in mean (SD) and qualitative data in percentage (%).

Variable	Category	Frequency	Percentage (%)
Age	Mean (SD)	33.10 (8.93)	-
	Range (Min-Max)	19–50 years	-
Sex	Male	37	52.9%
	Female	33	47.1%
Perforation size	Small	15	21.4%
	Medium	27	38.6%
	Large	26	37.1%
	Subtotal	2	2.9%
Affected side	Left	40	57.1%
	Right	30	42.9%

**Figure 1:**
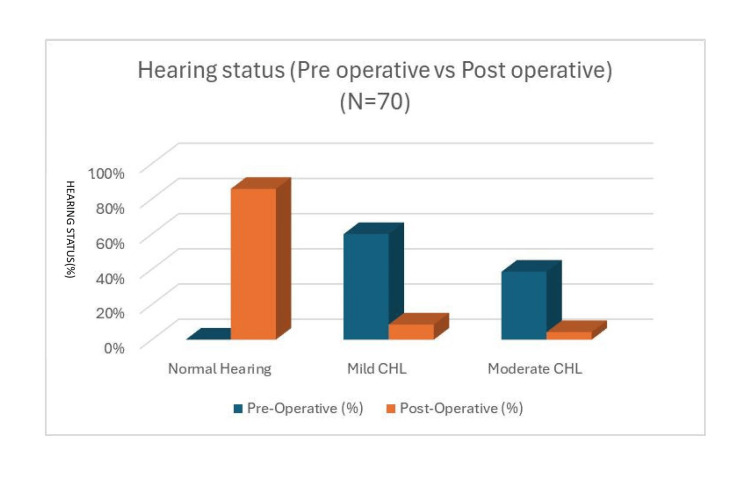
Distribution of study participants according to improvements in hearing pre- and postoperatively (N = 70) CHL: conductive hearing loss.

Preoperatively, no patients had normal hearing, with 60% having mild and 38.6% having moderate conductive hearing loss (CHL). Postoperatively, 85.7% achieved normal hearing, with mild CHL reduced to 8.6% and moderate CHL to 4.3%, a statistically significant improvement (χ² = 106.20, p = 0.001) (Figure 1).

Graft uptake was successful in 94.3% of cases. The AB gap improved significantly from 28.85 ± 7.99 to 12.42 ± 7.26, with a mean gain of 16.50 ± 7.77 (t = 17.603, p = 0.001).

**Figure 2:**
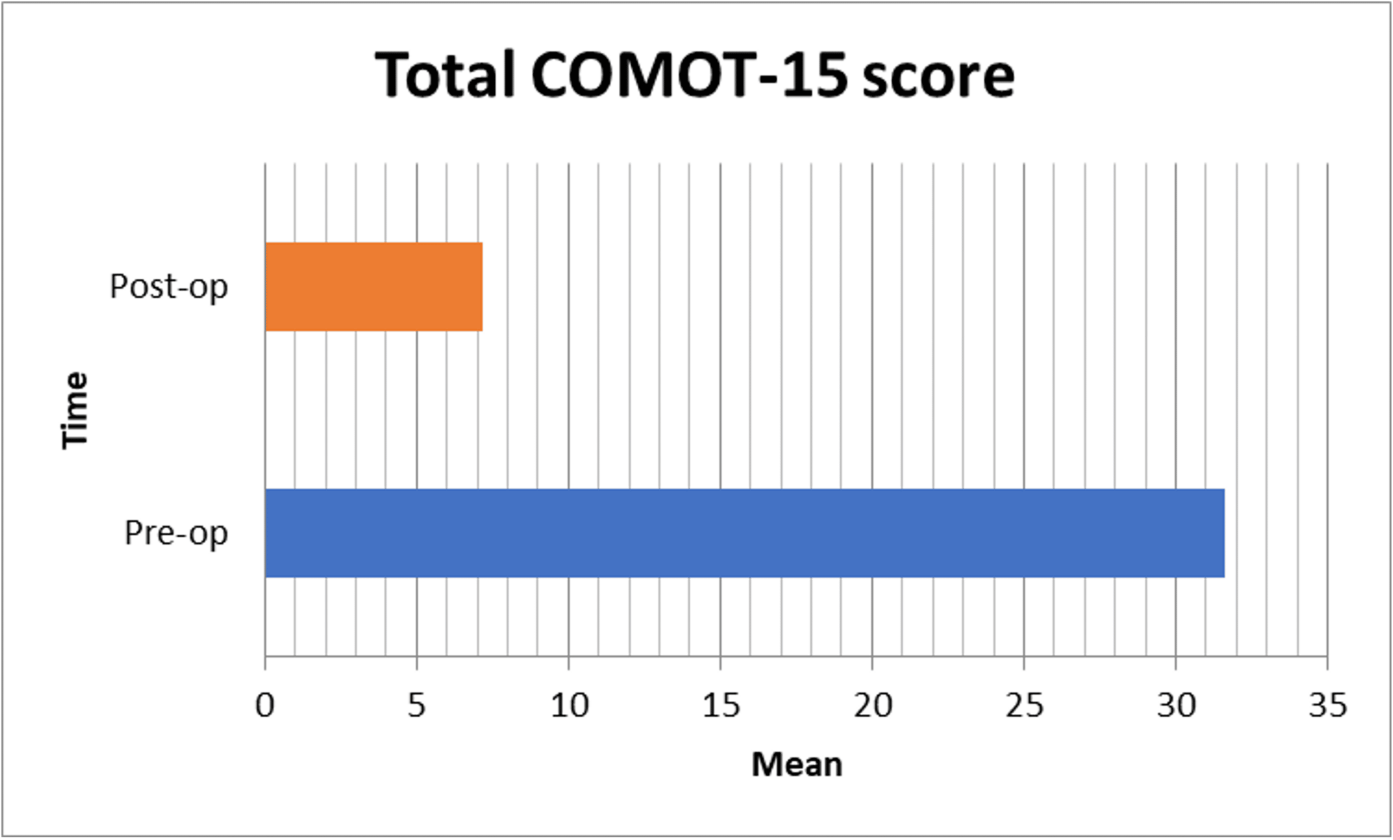
Distribution of study participants according to COMOT-15 scores pre- and postoperatively (N = 70) COMOT-15: Chronic Otitis Media Outcome Test 15.

The COMOT-15 scores improved significantly from 31.61 preoperatively to 7.18 postoperatively (p = 0.001) (Figure 2).

Psychological well-being scores decreased significantly from 11.92 ± 1.67 to 0.77 ± 2.49 (t = 31.997, p = 0.001). Severe ear discharge decreased significantly from 37.1% to 4.3% (χ² = 125.384, p = 0.001), and all patients reported no earache, pressure, or headache postoperatively, with statistically significant improvements (earache: χ² = 23.33, p = 0.001; ear pressure: χ² = 19.35, p = 0.001; headache: χ² = 8.485, p = 0.006).

Self-reported hearing loss and speech understanding difficulties improved significantly, with 85.7% reporting no issues postoperatively (hearing loss: χ² = 107.874, p = 0.001; speech from a distance: χ² = 112.879, p = 0.001; speech in noisy surroundings: χ² = 118.139, p = 0.001; simultaneous speech: χ² = 121.456, p = 0.001).

Emotional impacts like depression, embarrassment, and fear of worsening ear problems also decreased significantly (depression: χ² = 125.882, p = 0.001; embarrassment: χ² = 140.00, p = 0.001; fear of worsening: χ² = 94.490, p = 0.001). Overall quality of life improved significantly, with 85.7% reporting no issues postoperatively (χ² = 116.923, p = 0.001). Doctor visits increased significantly, with 77.1% requiring more than four visits postoperatively (χ² = 103.423, p = 0.001) (Table 2).

**Table 2:** Responses to COMOT-15 questions and hearing outcomes (N = 70) AB gap: air-bone gap. Chi-squared test was used to test statistical significance. COMOT-15: Chronic Otitis Media Outcome Test 15, CHL: conductive hearing loss.

Outcome	Pre-Op	Post-Op	Test Statistics	p-value
Hearing loss	Normal: 0%	Normal: 85.7%	X² = 106.20	0.001
	Mild CHL: 60%	Mild CHL: 8.6%		
	Moderate CHL: 38.6%	Moderate CHL: 4.3%		
	Moderately Severe: 1.4%	Moderately Severe: 1.4%		
Graft uptake	-	Yes: 94.3%	-	-
		No: 5.7%		
AB Gap (Mean ± SD)	28.85 ± 7.99	12.42 ± 7.26	t = 17.603	0.001
Gain in AB gap (Mean ± SD)	-	16.50 ± 7.77	-	-
COMOT-15 questions				
Discharge from the ear	No issue: 0%	No issue: 94.3%	X² = 125.384	0.001
	Severe issue: 37.1%	Severe issue: 4.3%		
Earache	No issue: 71.4%	No issue: 100%	X² = 23.33	0.001
	Severe issue: 1.4%			
Ear pressure/fullness	No issue: 75.7%	No issue: 100%	X² = 19.35	0.001
	Severe issue: 1.4%			
Tinnitus (ringing in the ear)	No issue: 100%	No issue: 100%	-	-
Headache	No issue: 88.6%	No issue: 100%	X² = 8.485	0.006
	Severe issue: 0%			
Hearing loss (self-reported)	No issue: 0%	No issue: 85.7%	X² = 107.874	0.001
	Severe issue: 27.1%	Severe issue: 4.3%		
Difficulty understanding speech from a distance	No issue: 0%	No issue: 85.7%	X² = 112.879	0.001
	Severe issue: 50%	Severe issue: 4.3%		
Difficulty understanding speech in noisy surroundings	No issue: 0%	No issue: 85.7%	X² = 118.139	0.001
	Severe issue: 68.6%	Severe issue: 4.3%		
Difficulty understanding simultaneous speech	No issue: 0%	No issue: 85.7%	X² = 121.456	0.001
	Severe issue: 68.6%	Severe issue: 4.3%		
Hearing loss causes depression/sadness	No issue: 0%	No issue: 94.3%	X² = 125.882	0.001
	Severe issue: 4.3%	Severe issue: 0%		
Fear of misunderstanding others	No issue: 0%	No issue: 94.3%	X² = 125.524	0.001
	Severe issue: 22.9%	Severe issue: 0%		
Hearing loss causes embarrassment	No issue: 0%	No issue: 100%	X² = 140.00	0.001
	Severe issue: 10%			
Fear of ear problems worsening	No issue: 8.6%	No issue: 90%	X² = 94.490	0.001
	Severe issue: 17.1%	Severe issue: 4.3%		
Overall impact on quality of life	No issue: 0%	No issue: 85.7%	X² = 116.923	0.001
	Severe issue: 31.4%	Severe issue: 0%		
Frequency of doctor visits	1–2 visits: 50%	3–4 visits: 22.8%	X² = 103.423	0.001
	3–4 visits: 50%	>4 visits: 77.1%		

Among patients with hearing improvement, 9.1% (6 out of 66) showed minimal mental improvement, while 90.9% (60 out of 66) demonstrated marked mental improvement. Among patients with no hearing improvement, 100% (4 out of 4) showed minimal mental improvement, and none exhibited marked mental improvement (Table 3).

**Table 3:** Association of hearing improvement with mental health improvement (N=70) Chi-squared test was used to test statistical significance.

Variable	Minimal mental improvement	Marked mental improvement	Total	X²	P value
Hearing improvement	6(9.1%)	60(90.9%)	66	25.5	<0.001
No hearing improvement	4(100%)	0(0%)	4		

**Figure 3:**
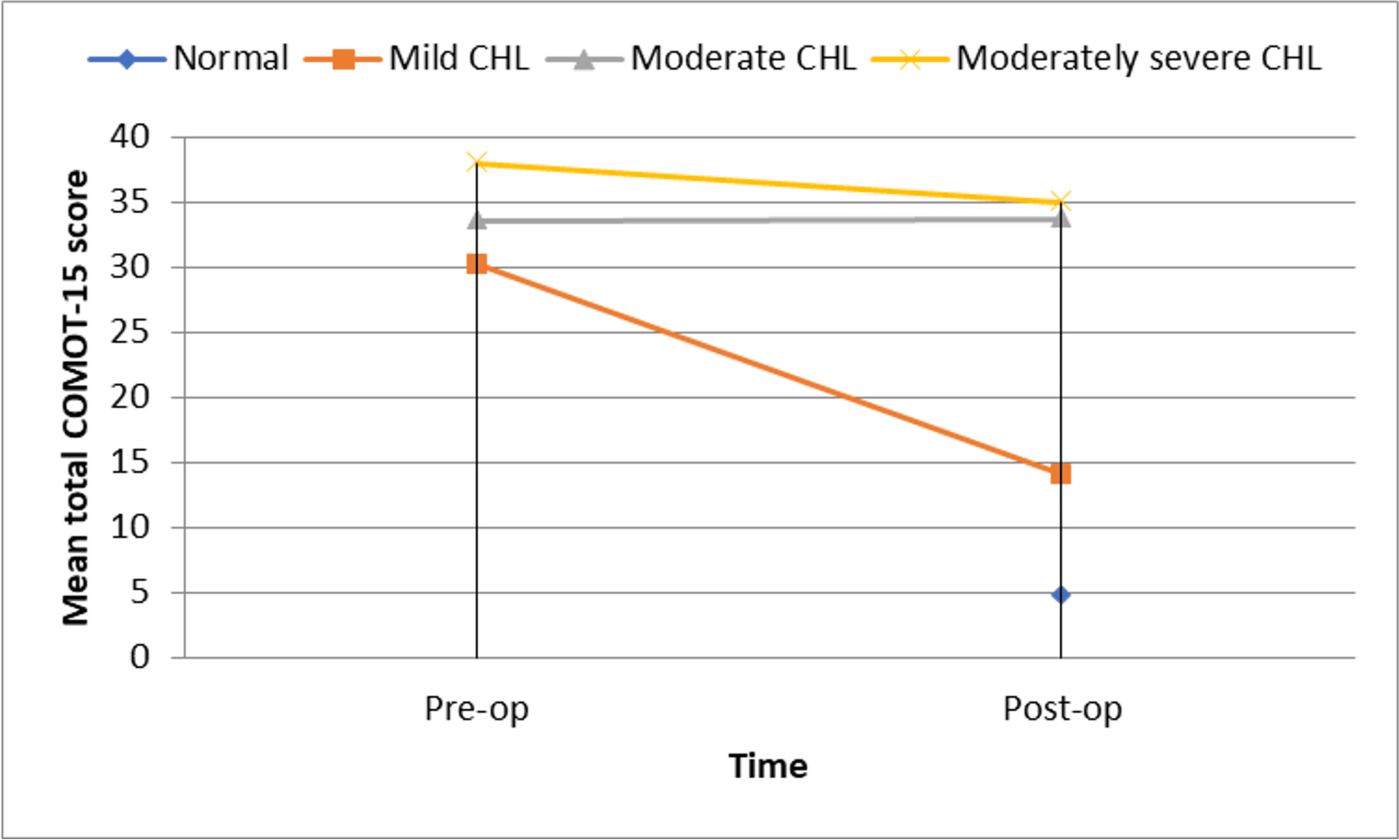
Distribution of patients with respect to total COMOT-15 score by time and hearing loss on PTA COMOT-15: Chronic Otitis Media Outcome Test 15, CHL: conductive hearing loss, PTA: pure-tone audiometry.

The association between hearing improvement and mental improvement was statistically significant, with a chi-square value of 25.5 and a p-value of less than 0.001 (Figure 3).

## Discussion

Our study is a hospital-based prospective investigation conducted over 18 months, involving 70 patients with unilateral mucosal COM undergoing Type I tympanoplasty. The primary focus was to evaluate QOL using the COMOT-15 questionnaire preoperatively and four months postoperatively. While surgical outcomes like graft uptake and hearing improvement are well documented, the QOL aspect in COM patients has been understudied. This study aimed to bridge this gap by assessing the subjective improvements in HR-QOL postsurgery.

The mean age of the participants in our study was 33.10 years, which aligns with findings from other studies. For instance, Devi et al. reported a mean age of 27 ± 8.70 years, while Evman et al. and Ali et al. reported mean ages of 33.47 ± 12.23 years and 28 ± 9 years, respectively [7-9]. In terms of sex distribution, 52.9% of our patients were male and 47.1% were female. Our findings are consistent with Gupta et al., who also reported a higher prevalence of COM in males (54%) [10].

In our study, medium-sized perforations (38.6%) were the most common, followed by large perforations (37.1%) and small perforations (21.4%). Subtotal perforations were rare (2.9%). These findings are consistent with Gupta et al., who reported central perforations in 76% of cases, and Bhatia et al., who observed central perforations in 75% of patients [10,11].

Regarding laterality, 57.1% of our patients had left-sided disease, while 42.9% had right-sided involvement. This contrasts with studies like Rasouli et al., where right-sided perforations were more common (74.6%), and Pragya Singh et al., who reported bilateral involvement in 32.9% of cases [12,13]. The predominance of left-sided disease in our study may be due to anatomical or environmental factors, though further research is needed to confirm this trend.

Preoperatively, none of our patients had normal hearing, with 60% exhibiting mild conductive hearing loss (CHL) and 38.6% moderate CHL. Postoperatively, 85.7% achieved normal hearing, with mild CHL reduced to 8.6% and moderate CHL to 4.3%. These improvements were statistically significant (p = 0.001). The air-bone (AB) gap also improved significantly, from a mean of 28.85 dB preoperatively to 12.42 dB postoperatively, with a mean gain of 16.50 ± 7.77 dB. These findings are consistent with Prakash Handi et al., who reported mild to moderate hearing loss in 83% of cases, and Khurshid et al., who observed similar degrees of hearing loss [14,15].

In our study, 94.3% of patients had successful graft uptake, while 5.7% experienced graft failure. This high success rate aligns with Devi et al., who reported graft uptake in 97% of cases by four weeks, and Bhatia et al., who observed successful graft uptake in 82% of cases at six months [7,11]. These results underscore the efficacy of Type I tympanoplasty in achieving durable tympanic membrane repair.

The COMOT-15 scores improved significantly from 31.61 preoperatively to 7.18 postoperatively (p = 0.001). This improvement was observed across all domains, including ear discharge, earache, ear pressure, headache, and self-reported hearing loss. Notably, psychological well-being scores decreased from 11.92 preoperatively to 0.77 postoperatively, indicating a marked reduction in the psychological burden associated with COM. These findings are consistent with Nisar et al., who reported significant improvements in COMOT-15 scores postsurgery, and Setiawan et al., who used the COMOT-15 scale to assess QOL in COM patients [16,17].

Our study found a statistically significant association between hearing improvement and mental health. Among patients with hearing improvement, 90.9% (60 out of 66) showed marked mental improvement, while only 9.1% (6 out of 66) showed minimal improvement. In contrast, all patients without hearing improvement (100%, 4 out of 4) exhibited minimal mental improvement. This association was statistically significant (χ² = 25.5, p < 0.001). These findings highlight the profound impact of hearing restoration on psychological well-being, emphasizing the importance of addressing both clinical and QOL aspects in COM management.

Despite the valuable insights provided by this study, certain limitations should be acknowledged. First, the study was conducted at a single hospital, which may limit the generalizability of the findings to broader populations or different healthcare settings. Second, the follow-up period was limited to four months postoperatively, which may not fully capture long-term outcomes or late complications. Third, the use of a single questionnaire (COMOT-15) for QOL assessment, while validated, may not encompass all aspects of patients’ psychosocial experiences. Additionally, factors such as socioeconomic status, education level, and occupation, which could influence both QOL and access to care, were not extensively analyzed. Finally, as with any self-reported measure, there is the potential for response bias in the COMOT-15 scores. Further, the exclusion of patients with comorbidities such as diabetes and those with squamosal chronic otitis media (COM) introduces selection bias, limiting the generalizability of findings to a relatively healthy subset of individuals with mucosal disease. Also, the absence of blinding for both patients and assessors poses a risk of bias, particularly given the use of subjective outcome measures such as the COMOT-15. Future multicenter studies with longer follow-up and a more comprehensive assessment of influencing factors are recommended to validate and expand upon these findings.

## Conclusions

This study reinforces the efficacy of Type I tympanoplasty as a definitive intervention for patients with unilateral mucosal chronic otitis media, demonstrating significant improvements in hearing, graft uptake, and health-related quality of life. The COMOT-15 questionnaire effectively captured the multidimensional benefits of surgery, including reductions in physical symptoms and psychological distress, as well as enhanced social functioning. The correlation between hearing improvement and mental well-being further emphasizes the broader therapeutic value of tympanoplasty beyond anatomical repair. These findings advocate for an integrated approach to COM management that prioritizes both clinical outcomes and patient-reported quality of life, thereby ensuring more holistic and impactful care.
